# Impact of ChatGPT on Diabetes Mellitus Self-Management Among Patients in Saudi Arabia

**DOI:** 10.7759/cureus.81855

**Published:** 2025-04-07

**Authors:** Turki M Alanzi, Wejdan Arif, Aldanah Alotaibi, Aasal Alnafisi, Raghad Alhwaimal, Nouf Altowairqi, Amal Alnifaie, Kadi Aldossari, Khulud Althumali, Nouf Alanzi

**Affiliations:** 1 Health Information Management and Technology, Imam Abdulrahman Bin Faisal University, Dammam, SAU; 2 Radiological Sciences, King Saud University, Riyadh, SAU; 3 Pharmacology, College of Pharmacy, Shaqra University, Riyadh, SAU; 4 Medicine, King Saud Bin Abdulaziz University for Health Sciences, Jeddah, SAU; 5 Pharmacology and Toxicology, College of Pharmacy, Jazan University, Jazan, SAU; 6 Pharmacology, College of Pharmacy, Shaqra University, Dawadmi, SAU; 7 Medicine, King Saud University, Riyadh, SAU; 8 Family Medicine, Prince Mansour Military Hospital, Taif, SAU; 9 Clinical Laboratory Sciences, Jouf University, Sakaka, SAU

**Keywords:** chatgpt, diabetes, healthcare, saudi arabia, self-management

## Abstract

Background: Diabetes mellitus (DM) is a chronic condition requiring continuous self-management to prevent complications. Artificial intelligence (AI)-driven tools like ChatGPT (OpenAI, Inc., San Francisco, CA, USA) offer potential support in education, monitoring, and decision-making. However, research on its effectiveness in DM self-management remains limited, particularly in Saudi Arabia, necessitating further investigation into its role and impact.

Purpose: This study aims to analyze the impact of ChatGPT on DM self-management.

Methods: A qualitative experimental design was adopted in this study. DM patients after interacting with ChatGPT for a week participated in the interviews, where their perceptions on its impact were recorded. A total of 25 DM patients participated in the study, whose results were analyzed using thematic analysis.

Results: The analysis of interview data revealed 11 themes related to the impact of ChatGPT on DM self-management, which included informational support, personalized recommendations, motivation and support, assistance in decision-making, offering self-care reminders, facilitating communication with healthcare providers, facilitating peer support, providing mental health support, tracking and monitoring, conducting health assessments, and education and awareness.

Conclusion: ChatGPT has a positive impact on DM self-management. However, further research is needed due to ChatGPT's novel nature for generalizing results and extending its applicability to other areas of healthcare.

## Introduction

Diabetes mellitus (DM) is a chronic metabolic disorder characterized by high blood sugar levels due to impaired insulin production or function. Uncontrolled blood sugar levels can result in serious health complications, including heart disease, stroke, kidney disease, nerve damage, and blindness [1-6]. DM self-management is essential for individuals living with DM, as it enables them to take control of their condition and reduce the risk of complications. Effective self-management involves lifestyle modifications such as maintaining a balanced diet, regular physical activity, routine blood sugar monitoring, adherence to prescribed medications, and stress management. By actively participating in their own care, individuals with DM can improve their health outcomes and quality of life.

Despite its importance, DM self-management presents several challenges. These include maintaining long-term compliance with treatment regimens, lack of knowledge and education about effective self-care practices, emotional distress associated with managing a chronic illness, financial burdens related to DM care, and social and cultural barriers [7-17]. Addressing these challenges requires continuous education, healthcare support, and innovative solutions to facilitate self-management practices.

With the increasing adoption of artificial intelligence (AI) in healthcare, AI-driven solutions like ChatGPT (OpenAI, Inc., San Francisco, CA, USA) offer new possibilities for improving DM self-management. ChatGPT, a large language model based on natural language processing and deep learning, has demonstrated capabilities in providing accurate, relevant, and well-structured responses to various queries [18-24]. Studies [25-28] have identified the significant relevance of ChatGPT application in healthcare but also suggested the need for further research to fully understand the impact of ChatGPT on patients' self-management practices in relation to various conditions. This study aims to explore the perceptions of patients in Saudi Arabia regarding the impact of ChatGPT on DM self-management, thereby contributing new insights into AI applications in chronic disease management.

Background

Globally, DM prevalence has been rising at an alarming rate. In 2021, approximately 537 million adults were diagnosed with DM, and this number is expected to increase to 783 million by 2045. Additionally, 541 million adults have impaired glucose tolerance (IGT), placing them at high risk of developing type 2 DM. The global prevalence was 9.8% in 2021, with three out of four cases occurring in low- and middle-income countries [18]. The mortality rate due to DM-related complications has also increased significantly, from 4.6 million in 2011 to 6.7 million in 2021, despite technological advancements in healthcare. Furthermore, the financial burden of care continues to escalate, with global-related health expenditures, amounting to $966 billion in 2021, projected to surpass $1 trillion by 2030 [19].

Existing literature highlights the importance of AI in healthcare, particularly in enhancing patient care, decision-making, and disease management. AI-driven applications have been successfully integrated into teleconsultations, diagnostics, and personalized treatment plans [20]. Recent advances in AI technologies, particularly in natural language processing and deep learning, have led to the development of models like ChatGPT. These models have been widely used for various applications such as language translation, text generation, and question-answering [21-23]. Studies have demonstrated that ChatGPT can provide accurate and relevant answers to health-related queries, assist in medical education, and support healthcare professionals in decision-making processes [24].

In the field of healthcare, ChatGPT has shown potential as a tool for teleconsultations and patient care [25,26]. Its ability to provide quick and accurate information can support teleconsultants in making informed decisions and offering better patient care [27]. Moreover, ChatGPT can assist patients by providing general health-related information, helping them understand their condition, and guiding them in self-management practices [28].

Although research has explored the role of AI in healthcare, limited studies have specifically investigated the impact of ChatGPT on patient self-management of DM. While existing studies recognize the relevance of ChatGPT in healthcare applications, they also emphasize the need for further research to evaluate its effectiveness in improving patient self-management practices across different conditions [29-33].

In Saudi Arabia, inadequate glycemic control remains a significant concern, particularly among individuals with type 2 DM. Studies [34,35] have reported a high prevalence of poor glycemic control (hemoglobin A1c (HbA1c) ≥7%), with several contributing factors hindering effective DM management. Unhealthy lifestyles, including sedentary behavior, high consumption of processed foods, and lack of physical activity, have been identified as major contributors to the rising prevalence of obesity and DM, making blood sugar regulation more challenging. Dietary habits also play a critical role, as research indicates that a significant portion of the Saudi population consumes fewer than one serving of fruits and vegetables per day, with unhealthy eating patterns being linked to higher HbA1c levels. Additionally, a lack of knowledge regarding HbA1c and essential DM management strategies, such as carbohydrate counting and structured self-monitoring, further complicates glycemic control.

While mobile health (mHealth) technologies offer potential solutions for DM self-management, several barriers hinder their effective adoption in Saudi Arabia. These include a shortage of mHealth expertise and human resources, inadequate funding and infrastructure investments, legal and privacy concerns, and organizational impediments within healthcare systems [36]. Addressing these challenges is crucial for improving health outcomes and promoting effective DM self-management in the region. These factors highlight the urgent need for effective, accessible, and personalized DM self-management solutions to improve health outcomes in Saudi Arabia.

Research gap and contribution

While prior research has acknowledged the potential of AI in healthcare, there remains a lack of empirical studies assessing the impact of AI-driven chatbots like ChatGPT on chronic disease self-management, particularly. In Saudi Arabia, where prevalence is significantly high, understanding how AI-powered solutions can enhance self-management is crucial. Existing knowledge does not adequately address patient perspectives on the usability, benefits, and limitations of ChatGPT for self-management.

This study aims to bridge this gap by exploring patient perceptions of ChatGPT's role in self-management in Saudi Arabia. By examining how patients engage with ChatGPT, the study will contribute to the broader discourse on AI applications in healthcare, providing insights into the feasibility, challenges, and potential benefits of AI-driven support systems for chronic disease management. The findings will inform healthcare practitioners, policymakers, and technology developers on how AI-powered tools can be optimized to support self-care, ultimately improving health outcomes and quality of life for patients.

## Materials and methods

Study settings and participants

This study adopted a qualitative-experimental design with a single-group pre-test/post-test approach to evaluate the impact of ChatGPT on self-management. The study was conducted at King Fahad University Hospital in Saudi Arabia, where outpatients were recruited during their outpatient visits. Participants were asked to interact with ChatGPT (free version 3.5) for one week to seek information about self-management.

To ensure structured engagement, participants were provided with guidelines on how to interact with ChatGPT, including sample queries related to diet, exercise, medication, blood sugar monitoring, and stress management. They were encouraged to ask both structured and unstructured questions in either Arabic or English. The interaction experience was unrestricted in terms of time and frequency, allowing participants to explore the tool in a manner that suited their individual preferences.

After one week, participants attended in-depth interviews where they discussed their experiences with ChatGPT. The interview data were analyzed using thematic analysis to identify recurring themes regarding the perceived impact of ChatGPT on self-management.

Recruitment and sampling

A purposive sampling strategy was used to recruit outpatients for the study. Initially, 48 patients were screened for their awareness of ChatGPT. Out of these, 27 participants who were familiar with ChatGPT and its functionalities agreed to take part in the study. However, two participants dropped out before the interviews, leaving a final sample size of 25. The sample size was justified based on qualitative research guidelines, which suggest that a sample of 20-30 participants is appropriate for studies using thematic analysis in semi-structured interviews [37]. This range allows for sufficient data saturation while maintaining feasibility in data collection and analysis. Additionally, previous studies in healthcare AI applications have used similar sample sizes to explore patient experiences and perceptions.

Questionnaire development

Semi-structured interviews are flexible in terms of conducting the interviews. It is important to note that in these types of interviews, there are no fixed set of questions that are designed before starting the interviews (structured interviews), but there are a few questions initially designed by the researchers. However, there is a flexibility to extend the existing questions or add new questions during the interview based on the responses of interviewees [38]. Accordingly, the interview questionnaire was designed by the authors, and it has four demographic-related questions including gender, age, education, and employment status. In addition, there are 30 questions related to the influence of ChatGPT on the participants' related knowledge and self-care and the changes they observed. The questions can be found in Appendix A. The interview questions were then translated into Arabic, using a professional translator. The translated questionnaire is validated by the two professors from the eHealth department at Imam Abdulrahman Bin Faisal University. A few changes related to grammar were suggested, and accordingly, the changes were made in the Arabic version. 

Data collection

The semi-structured interviews were conducted at the university hospital and conducted in Arabic language. They were audio-recorded. Each interview lasted for 40-60 minutes, and the average interview time was approximately 52 minutes. Interviews were audio-recorded with the consent of the participants.

Data analysis

The recorded interviews were transcribed verbatim into text documents using NVivo (Lumivero, Denver, CO, USA) to facilitate systematic data organization and analysis. Since the interviews were conducted in Arabic, the transcripts were professionally translated into English to ensure accuracy and consistency before analysis. A thematic analysis framework, as proposed by Braun and Clarke [39], was used to analyze the data, following a structured six-step process. First, researchers familiarized themselves with the data by reading and re-reading transcripts to gain an in-depth understanding. Next, open coding was conducted, where meaningful text segments related to self-management and ChatGPT's impact were manually coded in NVivo. These initial codes were then grouped based on conceptual similarities to form broader themes. The themes were subsequently reviewed for coherence and relevance to the study objectives, ensuring that they accurately represented participants' experiences. Each theme was clearly defined and named to capture its core meaning before being synthesized into a narrative format, supported by direct participant quotations to enhance validity. To ensure credibility and reliability, two independent researchers conducted the initial coding and theme development, with inter-coder reliability assessed through discussions to resolve any discrepancies. This rigorous analytical approach ensured consistency and depth in the interpretation of findings.

Ethical considerations

The ethical approval for the study was received from the Institutional Review Board of Imam Abdulrahman Bin Faisal University (approval number: IRB-2023-03-149). All standard ethical practices were followed in the process of data collection and analysis. The participants were fully informed about the nature of the study and were explained about their rights. The anonymity of the participants was ensured by using pseudo names for each interviewee. Participation was voluntary, and informed consent was obtained from all the interviewees before starting the interviews.

## Results

Characteristics of participants

Twenty-five DM patients participated in the interviews. Eleven participants were females and 14 participants were males. The demographic information of the participants is presented in Table 1. The detailed information of each participant is presented in Appendix B.

**Table 1:** Participants' demographics

Demographic data	Frequency
Gender	
Male	14
Female	11
Age	
18-30 years	12
31-40 years	9
41-50 years	4
Education	
Diploma	7
Bachelor's degree	13
Master's degree	4
PhD	1
Employment status	
Employed	19
Unemployed	6

Impact/influence of ChatGPT on DM self-management

**Figure 1:**
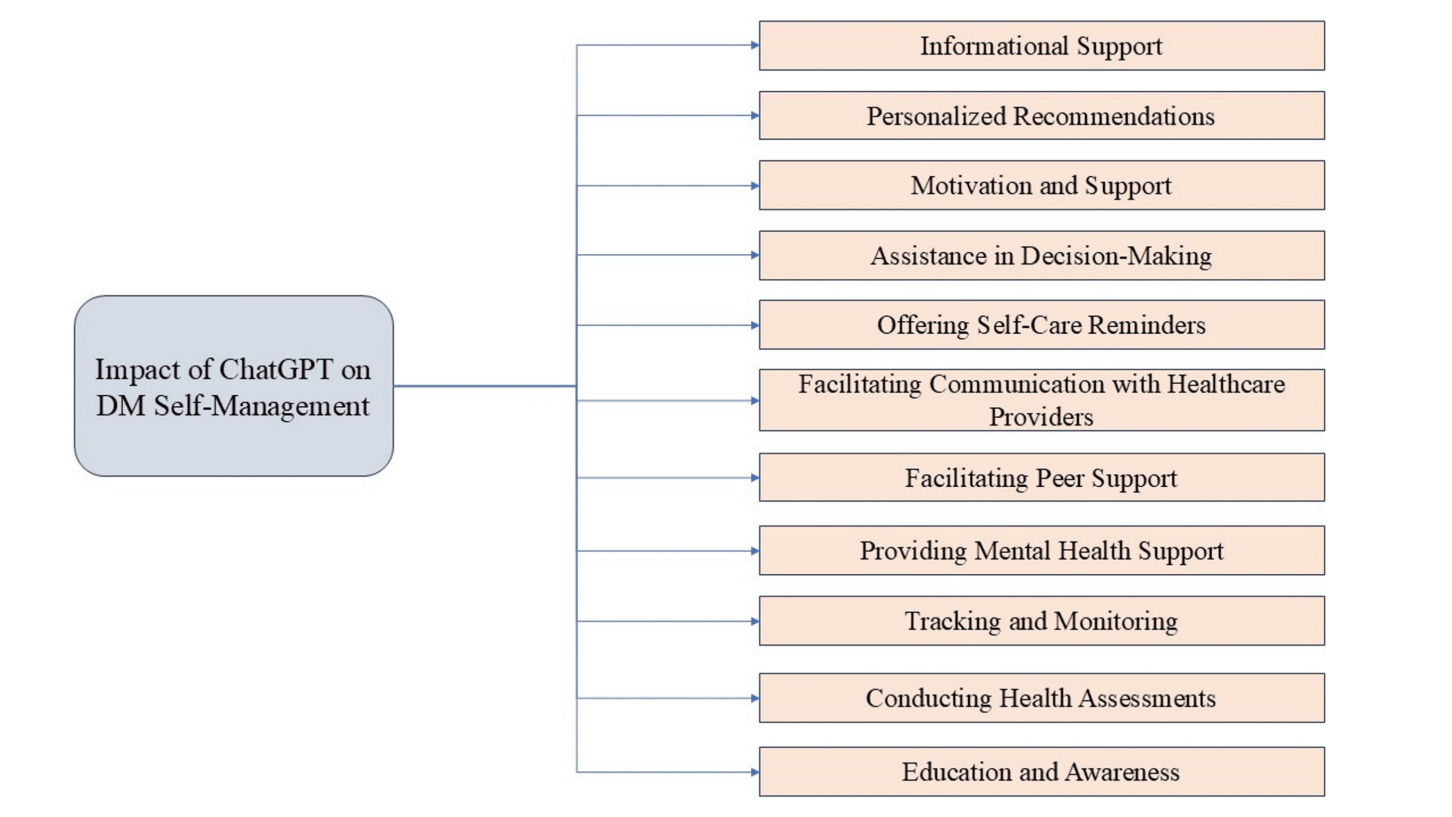
Themes related to the impact of ChatGPT on DM self-management DM: diabetes mellitus

The analysis of interview data revealed 11 themes (Figure 1) related to the impact of ChatGPT on DM self-management, which are discussed below:

Informational Support

One of the key impacts of ChatGPT as identified by all the participants was informational support. All the participants agreed that ChatGPT provides accurate and up-to-date information on self-care, including diet and nutrition, exercise, blood sugar monitoring, and medication management. Participants stated that detailed information on the risk factors such as alcohol intake, smoking, obesity, and reduced physical activity are a few examples of important information that they received while interacting with ChatGPT. For example, Interviewee 7 stated that "I was not aware about the importance of exercise for managing DM. However, after interacting with the application, I came to know that exercises can improve insulin sensitivity. Moreover, the application also presented different types of simple exercises such as stretching, cycling, dancing, and jogging. Brisk walking of 2000 to 4000 steps was suggested as it is suitable exercise for all those without ortho problems in the legs."

Furthermore, different types of diets and foods that are less in glucose were suggested by the application. It is interesting to note that a few new dishes according to the taste preferences of the patients were suggested by the application by using foods that are low in calories. Similarly, the types of exercises and the importance of monitoring were very well understood by the patients by interacting with ChatGPT. The application was identified to be beneficial for older patients (Interviewee 4, Interviewee 11, Interviewee 15) who are not aware of self-management care and also the complications of DM. For instance, Interviewee 15 stated that "I became aware that medication management is an important aspect of DM management because it can help to regulate blood sugar levels and reduce the risk of short-term and long-term complications. Also, by adopting lifestyle changes such as exercise, balanced diet, we can reduce the medication intake after consultation with the physician. However, the decision can be taken after monitoring the glucose levels for a period of time. The application also suggested real-time glucose monitoring patch powered by AI technologies, which can monitor the glucose levels 24×7 and change diet and medication accordingly."

In addition, Interviewee 4 stated that "I didn't know much about how different foods affect my blood sugar. ChatGPT explained the importance of the Mediterranean diet and suggested specific meals like grilled fish with olive oil and whole grains. This was very helpful because it aligned with my cultural food preferences."

Participants also reported that ChatGPT provided comprehensive information on self-management, including diet, exercise, blood sugar monitoring, and, most importantly, the complications. For example, Interviewee 16 stated that "The application helped me in learning about the importance of monitoring blood glucose levels. I got to know from the application that when blood sugar levels are too high, it can cause damage to organs and tissues over time, leading to long-term complications such as nerve damage, kidney damage, and eye damage. When blood sugar levels are too low, it can cause immediate symptoms such as dizziness, confusion, and sweating and can be potentially life-threatening."

Personalized Recommendations

ChatGPT offered personalized recommendations based on participants' specific needs, preferences, and lifestyle factors. For example, ChatGPT suggested meal plans that are tailored to an individual's dietary restrictions, cultural background, and food. Accordingly, Interviewee 5 stated that "When I asked a sample vegetarian diet, the application gave me a meal plan for breakfast, two-time snacks, and dinner. It recommended oatmeal along with nuts, berries, and non-dairy milk for breakfast; raw boiled vegetables for afternoon snacks; and, for lunch, items such as grilled vegetable sandwich on whole grain bread with avocado spread; Greek yogurt for evening snacks; and tofu stir-fry with brown rice and steamed broccoli for dinner. The meal plan was very much customized according to my requirements."

Furthermore, personalized recommendations were offered for exercise routines, notifications or messages for reducing stress, etc. This can be inferred from Interviewee 1's statement: "I have cardiac problems and also DM; as a result, I may have to be careful in doing physical exercises. When I asked the application, it initially suggested to consult physician, but also suggested some customized routine. It suggested low-intensity exercises such as walking or cycling and gradually increase the intensity and duration over time. In addition, it suggested low-impact exercises that are gentle on the joints and minimize the risk of injury, such as swimming, yoga, or tai chi."

In a similar context, Interviewee 8 stated that "The most interesting aspect is routine. It suggested five to 10 minutes for warm-up exercises such as brisk walking before indulging in any physical exercises and later cardiovascular exercises, such as yoga, tai chi, followed by cool-down exercise such as brisk walking."

ChatGPT also provided customized diet and exercise suggestions based on participants' needs, including cultural and religious considerations (e.g., fasting during Ramadan). Accordingly, Interviewee 7 stated that "When I asked for an exercise plan, ChatGPT suggested brisk walking after Iftar during Ramadan, instead of during fasting hours, which I found practical and manageable."

Motivation and Support

ChatGPT was identified to be providing motivation and support to participants with DM by offering positive reinforcement, reminding them of their goals, and providing encouragement to stick to their self-care routines. In this context, Interviewee 13 stated that "Whenever I mentioned my progress in blood sugar control, ChatGPT encouraged me and even celebrated my small successes. It made me feel like I was not alone in this journey."

This approach can help individuals stay motivated and committed to their DM management plan. ChatGPT helped motivate participants by offering positive reinforcement and goal-setting strategies. Accordingly, Interviewee 23 stated that "It encouraged me to set goals for self-care, such as changing and adapting to new diet regime, increasing physical activity, regular monitoring of blood sugar levels. The application motivated me to continue the self-practices by suggesting personalized recommendations."

Assistance in Decision-Making

ChatGPT assisted participants in making decisions about their self-care by analyzing data from their blood sugar readings, medications, and lifestyle factors. This helped them to make more informed decisions about their health and adjust their self-care practices accordingly. For instance, one participant used a continuous glucose monitoring (CGM) patch and provided daily readings to the application and asked for suggestions. In this context, Interviewee 15 stated that "I logged my blood sugar levels daily and asked ChatGPT if I needed to change anything. It suggested increasing fiber intake and monitoring for another few days before seeing my doctor. This helped me make informed decisions."

As the blood glucose levels reflected an increase continuously for three days, the application suggested a consultation with a doctor. Some participants used ChatGPT to understand their glucose fluctuations and decide whether to adjust their diet or consult a doctor. For some participants, ChatGPT supported in taking decisions in relation to physical activities. For instance, Interviewee 25 stated that "The categorization of different types of physical activities and exercises really helped me to better understand the need for structured exercise plans, and accordingly, I was able to make better decisions on selecting the type of exercise for a particular day."

Furthermore, the application provided a wide range of recommendations, enabling the participants to make informed and effective decisions in relation to self-management. In this context, Interviewee 21 stated that "I was given different recommendations and diet routines based on my likes and dislikes for food types. I informed that I like spicy foods. Based on my requirements, the application offered me different routines that included Mediterranean, DASH, and low-carbohydrate diets."

Offering Self-Care Reminders

While ChatGPT itself does not provide active reminders, participants learned about AI-integrated health apps that could help them schedule medication and glucose monitoring. This can help individuals stay on track with their self-care routines and prevent complications. As the participants used the ChatGPT web application, it informed participants that it could help them in reminding various medications. In this context, Interviewee 17 stated that "When I asked the application if it can remind me about medicine intake, it stated that it cannot, as it is an AI language model. However, it said that there are AI-based eHealth applications that uses its models which can provide recommendations and reminders by monitoring the blood glucose levels."

Facilitating Communication With Healthcare Providers

ChatGPT has the potential to support DM patients in improving communication with their healthcare providers, primarily by helping them prepare for medical consultations. For instance, Interviewee 14 stated that "Thanks to the study and the application. The application was very engaging, and it helped me in learning many new things about DM self-management. Earlier, I used to just follow what doctor says, and I don't know what to ask, as I was not aware about my disease. But now, I can ask many things like my exercise regime, diet plans, and many things that help me in better managing the blood glucose levels."

In a similar context, Interviewee 6 stated that "Before my last check-up, I asked ChatGPT what I should discuss with my doctor. It suggested asking about insulin dose adjustments and diet changes. I felt more confident in my appointment."

Several participants reported that ChatGPT enabled them to ask more informed questions about their treatment, diet, and exercise, leading to more productive discussions with their doctors. Accordingly, Interviewee 22 stated that "When I asked how can it help me in communicating with my healthcare provider, it stated that it can help them schedule appointments, provide updates on their condition, and ask questions about their treatment plan."

However, ChatGPT does not directly facilitate communication or integrate with eHealth applications, and any claims about appointment scheduling or provider interaction remain hypothetical rather than actual functionalities.

Facilitating Peer Support

ChatGPT played a role in helping participants discover online DM support groups, which allowed them to access community-based advice, shared experiences, and emotional support. In this context, Interviewee 18 stated that "I was informed by the application that it can be integrated with online platforms in order to facilitate DM patients to connect with one another and share their experiences."

Similarly, interviewee 11 stated that "I never knew there were Arabic-speaking DM support groups. ChatGPT recommended a few, and now I have a community to share experiences with."

However, while ChatGPT suggested relevant online communities, it did not directly connect patients or facilitate real-time engagement. Accordingly, Interviewee 10 stated that "I was asking the application for DM self-management groups. It provided many groups such as 'DM daily,' 'Beyond Type 1,' 'T1D Exchange.' It helped me to come in contact with many other DM patients by sharing original sources, links, and also shared many informational messages."

Providing Mental Health Support

ChatGPT provided mental health support to individuals with DM by offering coping strategies for managing stress, anxiety, and depression. This can be inferred from Interviewee 2's statement: "I was very stressed that I was diagnosed with DM at a very young age. Suddenly, I had to modify my diet, daily routine, and many activities, which made me to stand out among my friends. As a result, I had to undergo lot of stress. But ever since I started using ChatGPT, I realized the various ways it can help me to cope up with the emotional stress. It provides ideas of relaxation techniques such as yoga, meditation. It suggests me physical activities to indulge in which can help both my condition and stress."

This can help individuals with DM better manage their mental health and improve their overall well-being. Participants found emotional relief in interacting with ChatGPT, especially when struggling with stress or DM-related anxiety. Accordingly, Interviewee 9 stated that "Managing DM is stressful, but ChatGPT taught me deep breathing exercises and stress relief techniques. It even cracked a joke when I said I was feeling down!"

Tracking and Monitoring

ChatGPT can be used for information on tracking an individual's progress in managing their DM and providing feedback on their self-care practices. For instance, Interviewee 21 stated that "I did not know about CGM patches before using ChatGPT. It explained how they track glucose levels in real time. Now I use one, and it has made a huge difference in how I manage my DM."

This approach can help individuals identify areas for improvement and make necessary changes to their self-care routine. Accordingly, Interviewee 15 stated that "I started using a continuous glucose monitoring (CGM) device, and I would input my daily readings into ChatGPT to ask if my glucose levels were stable. When I noticed that my readings were consistently high in the mornings, ChatGPT explained the possibility of the dawn phenomenon and suggested adjusting my evening meal and consulting my doctor. This helped me understand my patterns better and make small changes to improve my blood sugar control."

ChatGPT also introduced participants to CGM devices and their benefits. For instance, Interviewee 24 stated that "I was not aware of the continuous glucose monitoring process. The application suggested me continuous glucose monitoring (CGM) device, which is inserted under the skin, usually on the abdomen or upper arm. The sensor monitors the glucose levels continuously and transmits the data to the receiver's device which can be accessed from the application. I contacted my physician about it and got a CGM patch for me, and now I can check my glucose levels in real time. Thanks to ChatGPT for providing such important information."

Conducting Health Assessments

ChatGPT can be a useful tool to conduct health assessments to identify an individual's risk for developing DM and provide personalized recommendations for preventing or managing the condition. However, ChatGPT does not diagnose or assess medical risk with clinical precision, and it only provides generalized risk estimates based on user inputs. For instance, Interviewee 17 stated that "ChatGPT asked me a series of questions about my lifestyle and gave me a risk score for developing complications. It made me realize I needed to be more proactive about my health."

This approach can help individuals take proactive steps to improve their health and prevent the onset of DM. ChatGPT helped some participants assess their DM risk by guiding them through online risk assessment tools. Accordingly, Interviewee 12 stated that "I asked the application if it can assess the risk of getting DM. It stated that it can, but it wanted me to answer few questions like age, eating habits, lifestyle, etc. I answered them, and it accurately identified that there is high risk for me to develop DM."

Education and Awareness

ChatGPT played a role in enhancing patient education by directing participants to credible DM resources such as the American Diabetes Association (ADA) and the World Health Organization (WHO). In this context, Interviewee 6 stated that "The application provided links to various educational resources from American Diabetes Association (ADA), Diabetes Self-Management Magazine, National Diabetes Education Program, which were helpful in recognizing the reliable educational resources. This helped me to stay away from the resources that provide mis-/disinformation about DM and its self-management process."

This function helped participants access reliable, evidence-based information, reducing their reliance on potentially misleading or unverified sources. Unlike the informational support theme, which focused on ChatGPT's ability to provide general DM-related guidance, this theme specifically highlights how ChatGPT linked users to external, authoritative sources for further learning. Several participants appreciated this feature, as it helped them distinguish between scientifically valid content and misinformation. Accordingly, Interviewee 8 stated that "ChatGPT recommended the American Diabetes Association's website and research articles. Now I know where to find reliable information instead of random social media posts."

## Discussion

The purpose of this study is to analyze the impact of a novel AI-based application called ChatGPT on DM self-management. The findings from the semi-structured interviews' analysis have indicated its impact in different areas of DM self-management, especially in relation to informational support, education and awareness, personalized recommendations, and motivation and support. ChatGPT was identified to be effective by all the participants in providing information and links to educational resources. This can help individuals with DM make informed decisions about their health and improve their self-care practices. Thus, ChatGPT can be an effective tool for addressing DM-related problems among people such as lack of knowledge and awareness [13] and to reduce financial burden [10,16] by improving self-care practices. These findings are further supported by studies [21-24] which revealed the efficiency of ChatGPT in providing accurate answers. Furthermore, the application can also help in preventing the spread and impact of health mis-/disinformation, which can have adverse results on the patients by providing accurate and reliable sources of information as identified from the findings. This is particularly relevant in Saudi Arabia, where studies [34-37] indicate that a significant portion of the population lacks awareness about effective DM management strategies, including carbohydrate counting and CGM. Given the rising prevalence of type 2 DM in the region, AI-driven solutions like ChatGPT could serve as an accessible educational resource, bridging gaps in DM knowledge.

It is commonly observed that patients tend to lose control and get out of track due to a lack of control and motivation, especially in relation to diets and managing various conditions [12,14,17]. However, controlling DM continuously is the only way to a better quality of life [8] and prevent many chronic diseases related to the kidneys, eyes, heart, and brain [1-6]. The findings revealed that ChatGPT is effective in motivating the patients for self-care by providing informational support. Similarly, AI-based applications and chatbots were found to be effective in improving patients' motivation and encouraging them to better manage their conditions [39-43]. This finding aligns with the need for culturally tailored motivational strategies in Saudi Arabia, where social and familial influences play a strong role in health behaviors. The inclusion of culturally relevant content, such as dietary recommendations aligned with traditional Saudi meals or exercise guidance adaptable to the local climate, could enhance ChatGPT's effectiveness in promoting self-management.

Findings suggested that ChatGPT can potentially provide personalized recommendations for patients, which can be a major leap forward in patient-centered healthcare if proven to be successful. Studies [44,45] have realized the potential of ChatGPT in providing personalized recommendations to an extent but suggested further research in this area for better analysis. For instance, ChatGPT was supportive in providing personalized diet and exercise plans for the participants in the study; however, in relation to medication or treatment, it suggested physician consultation, rather than a personalized medication. This indicates that language models like ChatGPT are still to be validated and verified for providing personalized advices and recommendations in certain aspects of healthcare activities.

One of the important influences of ChatGPT is that it involves patients in the DM self-management decision-making process in different activities. For instance, patients are compelled to make a decision on diet plans, exercise routines, and online consultations after receiving recommendations and suggestions from ChatGPT. Furthermore, increased awareness and knowledge of DM would also enable in facilitating better communication between patients and healthcare providers, as patients know their condition better than before. Although studies [46-48] have proved ChatGPT and similar AI technologies to be effective in clinical decision-making, there is a lack of evidence on such technologies supporting their assistance in patients' decision-making for DM self-management. In Saudi Arabia, healthcare accessibility varies by region, with urban centers having more specialized DM care facilities than rural areas. AI-driven tools like ChatGPT could provide additional support for individuals in remote locations by helping them prepare for medical consultations and formulate informed questions for their healthcare providers. This could be particularly beneficial for patients with limited access to endocrinologists or DM educators, enabling them to better navigate their treatment plans.

Based on the responses to these questions, ChatGPT can calculate an individual's risk of developing DM using established risk assessment tools such as the American Diabetes Association's DM Risk Test or the Finnish DM Risk Score. If the assessment indicates that the individual is at risk for developing DM, ChatGPT can provide recommendations for reducing their risk, such as making dietary and lifestyle changes, increasing physical activity, and scheduling regular medical check-ups. Although ChatGPT could provide emotional support for the patients by providing informational and educational support, its effectiveness in providing motivational support for DM patients is under-researched [49].

Managing DM involves emotional and psychological challenges alongside physical health concerns. While ChatGPT does not provide counseling or direct peer support, it can offer educational guidance and suggest coping strategies such as mindfulness exercises and relaxation techniques. AI-driven mental health tools have shown potential in reducing anxiety and depression symptoms [50], but ChatGPT's role remains limited to general support rather than personalized intervention. Given these constraints, individuals with DM should seek professional mental health services for comprehensive emotional support.

Findings revealed that AI-enabled ChatGPT applications are powerful technologies that can be used for accessing information in real time and can play an important role as an assistant to many patients. Informational and educational support for improving DM knowledge and awareness was identified to be one of the important impacts of ChatGPT, which can be linked to several other influences such as facilitating patients' communication with peers and healthcare providers, conducting health assessments, motivation and support, and improved decision-making. Due to its novel nature, there isn't enough evidence to generalize the impact of ChatGPT in various healthcare applications [51]. Therefore, the findings in this study can contribute to the literature gaps and aid healthcare decision-makers in effectively implementing such novel technologies in chronic patients' self-management practices. Furthermore, the findings in this study would enable researchers to better understand the strengths and weaknesses of such AI technologies' application in self-management approaches in healthcare.

This study has several limitations. Firstly, it used the free version of ChatGPT rather than an eHealth-integrated application for DM self-management. Given that ChatGPT lacks direct integration with healthcare systems, it could not provide personalized medication recommendations, automated reminders, real-time monitoring, or direct communication with healthcare providers, features that could enhance its practical impact if incorporated into an eHealth platform. Secondly, the study relied on self-reported data without clinical validation (e.g., HbA1c measurements), which limits the ability to assess actual health improvements. Additionally, the sample consisted predominantly of young, educated, and employed individuals, introducing a tech-savvy bias that may not reflect the experiences of older adults or those with lower digital literacy. Finally, the study employed a single-method qualitative approach, which, while valuable for capturing in-depth patient perspectives, does not provide quantitative insights into behavioral or clinical changes. Future research should explore mixed-methods approaches incorporating objective health metrics and a more diverse participant pool to enhance the generalizability of findings.

## Conclusions

The aim of the study is to analyze the impact of ChatGPT on DM self-management. The findings revealed a significant positive impact on DM self-management as ChatGPT was found to be effective in providing benefits such as educational support, personalized recommendations, motivational support, and decision-making. However, due to its novel nature, the impact of ChatGPT in healthcare is under-researched, and accordingly, there is a need to extend this study to various areas such as self-management practices, teleconsultations, and patients' outcomes.

## Appendices

Appendix A: Interview questionnaire

1. How long have you been diagnosed with diabetes, and what type of diabetes do you have?

2. How do you currently manage your diabetes?

3. Have you ever used a diabetes self-management app or tool before? If so, which one(s)?

4. How comfortable are you with technology, such as using a smartphone or computer?

5. Have you used ChatGPT before? If so, how would you describe your experience?

6. How frequently have you used ChatGPT to help manage your diabetes?

7. Have you found ChatGPT to be a helpful tool for managing your diabetes? If so, can you provide specific examples?

8. Has ChatGPT helped you with understanding your blood sugar levels or managing your medications?

9. How have you incorporated ChatGPT into your daily diabetes management routine?

10. Do you feel that ChatGPT has made a positive impact on your diabetes self-management overall? If so, how?

11. Have you noticed any changes in your blood sugar levels since using ChatGPT?

12. Have you been able to make any lifestyle changes or modifications to your diabetes management plan as a result of using ChatGPT?

13. Do you feel that ChatGPT has helped you better understand your diabetes diagnosis and how to manage it?

14. How easy or difficult has it been for you to communicate with ChatGPT about your diabetes management concerns or questions?

15. Have you shared any information or insights gained from ChatGPT with your healthcare provider? If so, what was their response?

16. Do you think that ChatGPT could be a helpful tool for other people with diabetes who are looking to improve their self-management practices?

17. Are there any additional features or functionalities that you would like to see added to ChatGPT to further support diabetes self-management?

18. How would you compare ChatGPT to other diabetes self-management tools or resources that you have used in the past?

19. Has using ChatGPT made you feel more empowered or in control of your diabetes management?

20. Would you recommend ChatGPT to other people with diabetes? Why or why not?

21. How much did you know about diabetes before using ChatGPT?

22. Have you learned anything new about diabetes through your interactions with ChatGPT? If so, can you provide an example?

23. Have you found ChatGPT to be a helpful tool for increasing your knowledge and awareness about diabetes?

24. How important is it for you to stay informed and up-to-date about diabetes and its management?

25. What sources do you typically rely on for information about diabetes?

26. Have you used ChatGPT to learn about the potential complications associated with diabetes?

27. Do you feel that ChatGPT has helped you better understand how to prevent or manage diabetes complications?

28. Are there any topics related to diabetes management or prevention that you would like to learn more about through ChatGPT?

29. Have you shared any information or insights gained from ChatGPT with family members or friends who may also be at risk for diabetes?

30. How has your overall understanding of diabetes and its management changed since using ChatGPT?

Appendix B: Participants' details

**Table 2:** Interviewees' details

Interviewee	Gender	Age	Education	Employment
Interviewee 1	Male	34	Diploma	Unemployed
Interviewee 2	Male	19	Bachelor's degree	Employed
Interviewee 3	Female	24	Diploma	Unemployed
Interviewee 4	Female	47	Master's degree	Employed
Interviewee 5	Male	26	Bachelor's degree	Employed
Interviewee 6	Male	35	Diploma	Employed
Interviewee 7	Male	22	Bachelor's degree	Unemployed
Interviewee 8	Female	37	Master's degree	Employed
Interviewee 9	Female	32	Bachelor's degree	Employed
Interviewee 10	Female	28	Bachelor's degree	Employed
Interviewee 11	Female	50	Diploma	Employed
Interviewee 12	Female	21	Diploma	Employed
Interviewee 13	Male	39	Bachelor's degree	Employed
Interviewee 14	Female	27	Bachelor's degree	Unemployed
Interviewee 15	Male	49	PhD	Employed
Interviewee 16	Female	22	Bachelor's degree	Employed
Interviewee 17	Male	24	Bachelor's degree	Unemployed
Interviewee 18	Male	31	Diploma	Employed
Interviewee 19	Female	33	Bachelor's degree	Employed
Interviewee 20	Male	27	Bachelor's degree	Employed
Interviewee 21	Male	36	Master's degree	Employed
Interviewee 22	Female	22	Bachelor's degree	Employed
Interviewee 23	Male	29	Bachelor's degree	Employed
Interviewee 24	Male	41	Diploma	Unemployed
Interviewee 25	Male	35	Master's degree	Employed
